# Celebrating motherhood after Fontan operation: a difficult and distant dream?

**DOI:** 10.1136/openhrt-2024-002911

**Published:** 2024-10-09

**Authors:** Justin Paul Gnanaraj, Steaphen Anne Princy

**Affiliations:** 1 Institute of Cardiology, Madras Medical College and Government General Hospital, Chennai, Tamil Nadu, India; 2 Department of Cardiology, Tamil Nadu Government Multi Super Speciality Hospital, Chennai, Tamil Nadu, India

**Keywords:** Fontan Procedure, Epidemiology, Heart Defects, Congenital

 The survival of children with single ventricle physiology has been bleak until the development of innovative surgical procedures that improved the survival to adulthood. In 1958, William Glenn performed the Glenn surgery, a partial palliative procedure that provided some relief. A decade later, in 1968, Francis Fontan, a French surgeon, revolutionised treatment by performing the first Fontan procedure, which completely redirected vena caval blood flow to the pulmonary artery, bypassing the right ventricle.^1^ The Fontan procedure has undergone numerous refinements over the years, with the most commonly performed modern variations being the lateral tunnel and extracardiac total cavopulmonary connection. These advances have significantly improved outcomes, with 30-year survival rates in Fontan palliation now approaching 85%.^2^ However, this improved survival comes at the cost of systemic venous hypertension, non-pulsatile pulmonary flow, a preload-deprived left ventricle and fixed cardiac output, all of which pose significant risks during pregnancy.

With the dawn of corrective surgeries for cyanotic congenital heart diseases, an increasing number of women began surviving into adulthood and seeking to have children. Pregnancy-related haemodynamic changes confer a considerable burden on the Fontan heart and this population is likely to have maternal cardiovascular and obstetric morbidity. Historically, women who had a Fontan operation were advised to refrain from getting pregnant. However, this stance began to shift with several case reports and a study by Mary Canobbio and colleagues,^3^ which documented 33 pregnancies in 21 women with Fontan palliation, showing favourable maternal outcomes. The published data are reassuring that patients with Fontan physiology tolerate pregnancy much better than expected, and no maternal deaths have been reported in the subsequent publications to date. However, it is pertinent to recognise that there is potential for publication bias in reporting positive outcomes.

In this issue of *Open Heart*, Wazni *et al*^4^ retrospectively analysed 44 pregnancies in 20 women with single ventricle physiology treated from a single centre between 2005 and 2023, aiming to identify predictors of spontaneous pregnancy loss (SPL). Of these women, all but two had undergone the Fontan procedure, with Glenn and Waterston shunts performed in the remaining two. Noteworthy findings included moderate to severe atrioventricular (AV) valve regurgitation in 70% of patients, right ventricular (RV) morphology in 50%, a history of atrial arrhythmias in 80%, Fontan-associated liver disease in 75% and prepregnancy hypertension in 60%. A high proportion of patients were on antiarrhythmic (75%) and antihypertensive (75%) medications.

The path to motherhood for women with Fontan circulation is fraught with significant challenges. Although the live birth rate reported in this study was 45%, this figure should not be interpreted as the general likelihood of any woman with Fontan palliation achieving a live birth. These women face significant reproductive issues, including infertility, menstrual irregularities, recurrent miscarriages and pregnancy complications. Fertility onset is often delayed, with a higher rate of infertility largely attributed to hypoxia and clotting abnormalities.^5^ However, the current study does not provide the total number of post-Fontan women of childbearing age seen at the centre during the study period, making actual fertility rate unclear. Existing literature reports very low fertility rates in Fontan patients, ranging from 16% to 18%.^3 6^ Even among those who became pregnant, 21% had required infertility therapy, according to the study by Gouton *et al*.^7^ The authors observed a 48% incidence of SPL (21 out of 44) and a live birth rate of 45% (20 out of 44), emphasising the high rate of unsuccessful pregnancies despite conception. The reported SPL rates in the literature vary widely from 27% to 69%. Consequently, the live birth rates are similarly low, ranging from 24% to 66%.^7 8^

The authors identified RV morphology of the systemic ventricle, moderate to severe AV valve regurgitations and lower systemic arterial oxygen saturation (SaO_2_) in the first trimester as predictors of SPL. The difference in SaO_2_ between live births and SPLs was 3%. Although no significant drop in SaO_2_ was observed during pregnancy, there was a postpartum decrease of 1.8% in the live birth group and 1.5% in the SPL group, highlighting the importance of vigilant postpartum care. The study, however, does not provide prepregnancy data on the response of SaO_2_ to exercise, which might have shown a stronger association with SPL. A larger study of 78 women with Fontan circulation did not find similar association of SPL with SaO_2_.^8^ In another study of 116 pregnancies, all 8 pregnancies with SaO_2_ <85% resulted in SPL. However, among the remaining 106 pregnancies with SaO_2_ ≥85%, no significant correlation with miscarriage was found.^5^

**Figure 1:**
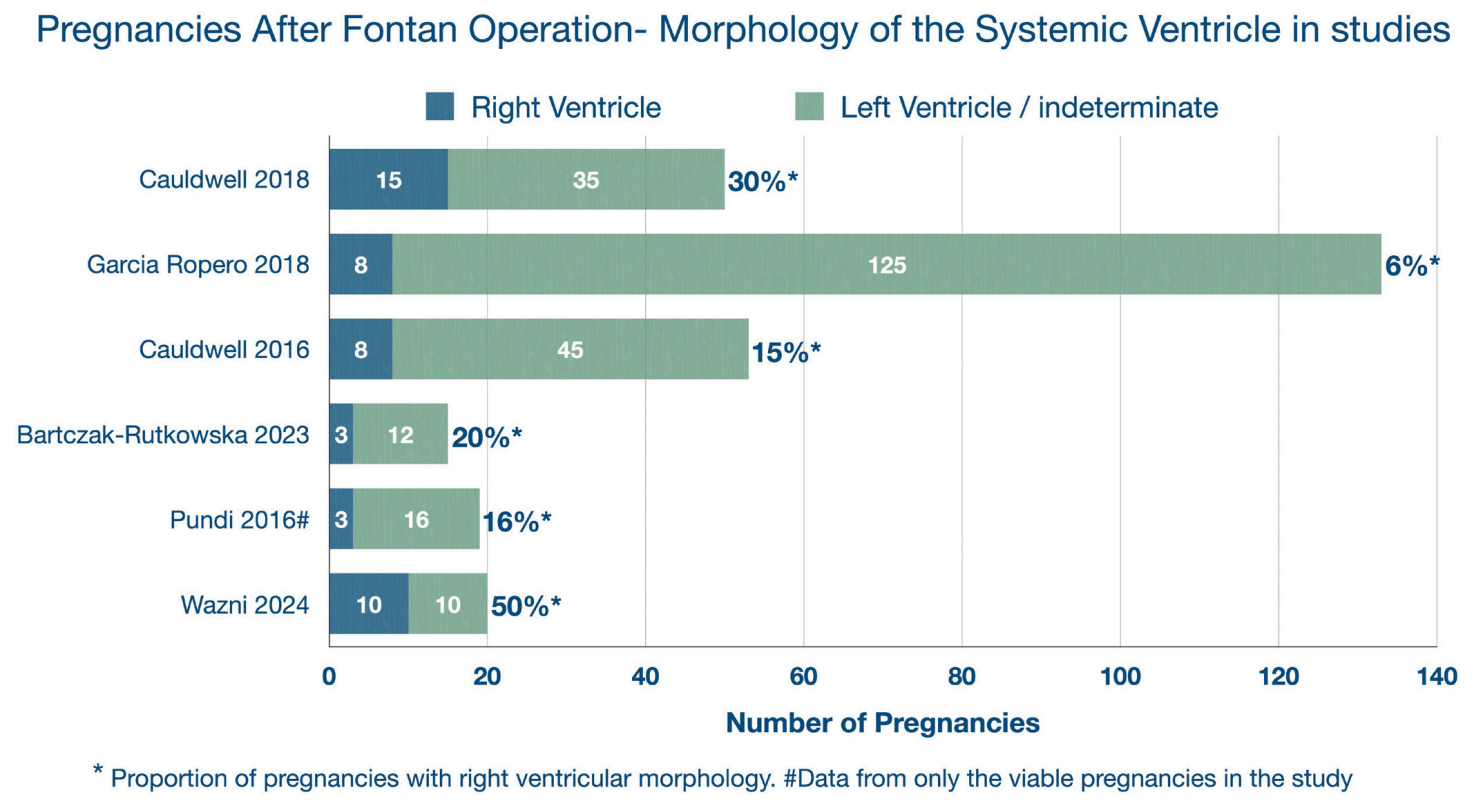
Pregnancies after Fontan operation: morphology of systemic ventricle in studies.

The high proportion of patients with RV morphology (50%) in this study is notable. Previous studies in women with single ventricle physiology have reported much lower rates, ranging from 6% to 30%^59^,^12^ (see figure 1). Pundi *et al*^11^ found 16% and 26% RV morphology among those with and without viable pregnancies, respectively, but did not identify RV morphology as a predictor of adverse pregnancy outcomes. Earlier studies have identified that prepregnancy functional class and systemic ventricular systolic function are associated with SPL.^8^ The authors did not provide data on prepregnancy functional class. Additionally, systemic ventricular ejection fraction was treated as a binary variable with a cut-off of 50%. However, treating systemic ventricular ejection fraction as a continuous variable might have revealed a stronger association with SPL. The study also reports a high prevalence of smoking (45%) and alcohol use (50%), both of which are known risk factors for SPL. However, these variables were not included in the analysis as potential predictors. Similarly, factors such as prepregnancy hypertension (60%) and substance abuse (20%) were also not included in the predictor analysis. Including these variables might have altered the final list of predictors.

It is concerning that only 22% of women who became pregnant after Fontan palliation had a documented preconception counselling visit, which is far from ideal. Preconception counselling is associated with improved pregnancy outcomes in women with congenital heart diseases.^13^ In contrast, two other studies have reported a much higher rate of preconception counselling at 82%.^5 8^ Effective preconception counselling provides an opportunity to optimise haemodynamics and overall health. This includes managing body weight, implementing exercise training and cardiac rehabilitation, correcting anaemia and iron deficiency, closing fenestrations, optimising cardiovascular risk factors, advising against substance use, and managing arrhythmias through electrophysiological studies and radiofrequency ablation, among other interventions.

As with other studies, there was no maternal mortality reported in this cohort. It is possible that SPL may act as a protective mechanism for mothers with suboptimal Fontan haemodynamics, who may not be able to safely tolerate the physiological demands of full-term pregnancy, delivery and the postpartum period.

Future prospective multicentre studies involving high-volume Fontan centres worldwide should be designed. These studies should assess the relationship between objectively measured functional capacity, resting SaO_2_, exercise-induced drops in SaO_2_, anaemia, iron deficiency and SPL, among other factors.

Despite significant advances in surgical techniques, women who survive to childbearing age after undergoing the Fontan procedure continue to face formidable obstacles to motherhood. The complex interplay between the physiological and haemodynamic changes of pregnancy and the Fontan circulation results in very low fertility rates, along with an increased risk of SPL, preterm birth, neonatal complications and maternal complications.

Optimising haemodynamic and physiological status during the preconception period, along with careful management of the delicate balance between thrombosis and bleeding throughout pregnancy, is crucial to achieving a successful live birth. For women who had a Fontan operation, the prospect of a successful pregnancy remains a challenging and often distant goal.
